# The Moderating Effect of Resilience on Mental Health Deterioration among COVID-19 Survivors in a Mexican Sample

**DOI:** 10.3390/healthcare10020305

**Published:** 2022-02-05

**Authors:** Héctor Raúl Pérez-Gómez, Esteban González-Díaz, Marta Herrero, Fabiola de Santos-Ávila, José Luis Vázquez-Castellanos, Pedro Juárez-Rodríguez, Bernardo Moreno-Jiménez, Rosa Martha Meda-Lara

**Affiliations:** 1 Centro Universitario de Ciencias de la Salud, Departmento de Clínicas Medicas, División de Disciplinas Clínicas, Universidad de Guadalajara, Guadalajara 44340, Mexico; hectorraul.perez@cucs.udg.mx; 2 Centro Universitario de Ciencias de la Salud, Departmento de Clínicas Médicas, Instituto de Patología Infecciosa y Experimental, Universidad de Guadalajara, Guadalajara 44340, Mexico; 3 Departamento de Psicología Social y del Desarrollo, Universidad de Deusto, 48007 Bilbao, Spain; m.herrero@deusto.es; 4 Centro Universitario de Ciencias de la Salud, Departamento de Disciplinas Filosófico, Metodológico e Instrumentales, Universidad de Guadalajara, Guadalajara 44340, Mexico; fabiola.desantos@academicos.udg.mx; 5 Centro Universitario de Ciencias de la Salud, Departamento de Salud Pública, Instituto Regional de Investigación en Salud Pública, Universidad de Guadalajara, Guadalajara 44340, Mexico; jose.vcastellanos@academicos.udg.mx; 6 Centro Universitario de Ciencias de la Salud, Departamento de Psicología Básica, Universidad de Guadalajara, Guadalajara 44340, Mexico; rosa.meda@academicos.udg.mx; 7 Facultad de Psicología, Universidad Autónoma de Madrid, 28049 Madrid, Spain; bernardo.moreno@uam.es

**Keywords:** resilience, depression, anxiety, stress, traumatic impact of the event, COVID-19, survivors

## Abstract

Resilience has been reported to be a protective psychological variable of mental health; however, little is known about its role in COVID-19 survivors. Thus, in this study, we aimed to evaluate the levels of depression, anxiety, stress, traumatic impact, and resilience associated with COVID-19, as well as to investigate the role of resilience as a moderating variable. A sample of 253 participants responded to an online survey; all were previously diagnosed with COVID-19 by a nasopharyngeal swab RT-PCR test, were older than 18 years, and signed an informed consent form. Significant negative correlations were found between resilience and the mental health variables. Higher resilience was significantly related to a lower impact of the event, stress, anxiety, and depression when the number of symptoms was low. Only when the duration of COVID-19 was short and resilience levels were medium or high was psychological distress reduced. Moreover, resilience moderated the effects of COVID-19 on mental health, even if a relapse occurred. The results emphasize the need for interdisciplinary interventions aimed at providing COVID-19 patients with psychological and social resources to cope with the disease, as well as with probable relapses.

## 1. Introduction

Globally, there have been more than 250 million total cases of COVID-19, and over 5 million total deaths have been reported [1], indicating that a high percentage of people infected with SARS-CoV-2 recover from the disease. Given the experience gained from the pandemic outbreaks of Severe Acute Respiratory Syndrome (SARS) [2] and Middle East Respiratory Syndrome (MERS) [3], clearly showing that the mental health sequelae in survivors can be catastrophic and long-lasting, it is necessary to study the mental health of patients that have recovered from SARS-CoV-2 infection in order to plan, in advance, how to manage and mitigate the psychological consequences of the disease through timely interventions. In this respect, several experts have reported the possibility of a subsequent pandemic of mental disorders flowing from COVID-19 [4,5].

Follow-up studies conducted on recovered COVID-19 patients highlight that many survivors report clinically significant levels of depression (9–38%) [6,7,8,9,10,11], stress (7.5%) [7], and anxiety (12–42%) [6,7,8,9,10] weeks and even months after suffering from the disease.

Regarding the traumatic impact caused by the infection in Chinese survivors, Bo et al. [12] recorded a prevalence of significant symptoms of 96.2% in the participants evaluated prior to their discharge from hospital; in another short-term follow-up study, relevant symptoms were identified in 25.1% of the participants [10]. In a six-month follow-up study, the prevalence of post-traumatic stress symptomatology in survivors in Iran was 5.8% [13]. A recent meta-analysis reported the prevalence of post-traumatic stress symptomatology in 20% of COVID-19 survivors [14]. Moreover, Chamberlain et al. [15] highlighted the positive correlation between the severity of the disease and the presence of post-traumatic stress symptomatology, whereas Einvik et al. [16] indicated differences in the presence of psychological distress between those survivors who were hospitalized (9.5%) and those who were not (7%) in a Norwegian sample.

Resilience is defined as the ability of an individual to maintain psychological equilibrium in difficult situations [17] and is characterized as the adaptive capacity of an individual to cope with and overcome overwhelming problems or stressful events [18]. Resilience mostly originates from social contexts and from resources that favor the psychological processes necessary to handle adversity [19]. Resilience promotes adaptation, resistance, or the recovery of mental and physical health after a challenge [20].

The variability of responses to the COVID-19 pandemic has generated the need to study individual differences and to analyze the psychological processes of coping with adversity and trauma [21]. Within the framework of the study of stress coping [22] and of the capacity for development in the face of adversity [23], resilience has been described as a multidimensional self-regulatory resource [24] with at least two core components, flexibility to adverse change [25] and the capacity to recover [26]. The recent review by Zhang et al. [27] shows that resilience can be considered as a global indicator of response resources in the face of traumatic events and personal development capacity in stressful contexts. In the COVID-19 pandemic, several studies have analyzed the effects of resilience on mental health. According to Chan et al. [28], in both individualistic and collectivistic cultures, high levels of resilience buffer the association between traumatic events and the appearance of anxious and depressive symptomatology. Havnen et al. [29] consider that such buffering effects would result from the application of a set of coping resources appropriate to the traumatic aspects of the pandemic. In the same vein, Hou et al. [30] indicate that resilience acts as an adaptive factor in the face of traumatic events. In summary, resilience would act as a resource that expresses the ability to maintain mental health despite adversities and life cycle challenges.

Several studies have evaluated the resilience of populations during the COVID-19 pandemic [18,31,32,33,34]; however, a gap in knowledge exists regarding the moderating role of resilience in the mental health deterioration of COVID-19 survivors.

In this study, we aimed to evaluate the levels of depression, anxiety, stress, traumatic impact, and resilience, as well as to investigate the moderating effect of resilience on the impact of the disease on mental health among COVID-19 survivors, since reports of this nature are currently lacking in the studied population. This will contribute key information for decision making in terms of public policies on mental health and pandemics.

## 2. Materials and Methods

The questionnaire and the informed consent form were converted into an electronic format on the SurveyMonkey platform, and the e-survey was built upon the Checklist for Reporting Results of Internet E-Surveys (CHERRIES) [35], as previously described [33].

Data were collected from 3 January to 18 August 2021 in two public and one private hospital from Guadalajara, Mexico. A total of 253 COVID-19 survivors answered the survey. All participants included in the study had a positive diagnosis of COVID-19 confirmed by a real-time polymerase chain reaction (RT-PCR) nasopharyngeal swab test.

The survey contained the following questionnaires and psychological scales:

- Sociodemographic questionnaire: including data considered for categorizing the population of this study included gender, age, education, occupation, relationships, children and elderly in the family, and family size. The variables considered were those used in similar studies and that, in this study, could be associated with mental health.
- COVID-19 related symptomatology: including nine questions on physical health status and medical problems related to COVID-19 in the 14 days prior to the survey. Symptoms included: fever, cold, headache, muscle pain, cough, chills, shortness of breath, chest pain, fatigue, hypogeusia, hyposmia, dizziness, diarrhea, vomiting, rhinorrhea, and sore throat.
- Impact of Event Scale-Revised (IES-R): originally named Impact Event Scale (IES) [36], was comprised of 22 items: 7 measure intrusion, 8 measure avoidance, and 7 measure hyperactivation. The intrusion subscale includes intrusive thoughts, nightmares, intrusive feelings and imagery, and dissociative-like re-experiencing. The avoidance subscale measures behaviors such as numbing of responsiveness and the avoidance of feelings, situations, and ideas. The hyperactivation subscale measures feelings and behaviors like anger, irritability, hypervigilance, concentration problems, and heightened startle. Participants were asked to indicate how worrisome the coronavirus pandemic experience had been for them over the past seven days. A 5-point Likert scale was used to evaluate the intensity of the symptoms, from 0 = “not worried” to 4 = “extremely.” Global assessment of traumatic impact was used to meet the objectives. The Chilean version of the instrument developed by Caamaño et al. [37] was applied to the current traumatic event and was used with an internal consistency coefficient of 0.98. Results were evaluated using the total score and the following cut-off points: 0–23 = normal; 23–32 = mild traumatic impact; 33–36 = moderate traumatic impact; >37 = severe traumatic impact [38].
- Depression, Anxiety and Stress Scales (DASS-21) [39]: developed to assess and designate the most common symptoms of negative affectation: depression, anxiety, and stress. DASS-21 uses a 4-point Likert scale from 0 = “It has not happened to me,” to 3 = “This has happened to me frequently”. The Spanish version of Daza et al. [40] instrument was used in the present study, to show internal consistency coefficients: global = 0.96; depression scale = 0.93; anxiety scale = 0.86; stress scale = 0.91. Results from each scale are evaluated using the following cut-off points for Depression scale: 0–9 = normal, 10–12 = mild, 13–20 = moderate, 21–27 = severe, 28–42 = severe extreme; Anxiety scale: 0–6 = normal, 7–9 = mild, 10–14 = moderate, 15–19 = severe, 20–42 = severe extreme; Stress scale: 0–10 = normal, 11–18 = mild, 19–26 = moderate, 27–34 = severe, 35–42 = severe extreme [34].
- Connor-Davidson Resilience Scale (CD-RISC) [17] in its 10-item version [41]: Used in other collective disaster situations for the assessment of resilience [42]. It uses a 5-point Likert scale from 0 = ‘totally disagree’ to 4 = ‘totally agree’. With an internal consistency coefficient of 0.92. Results were evaluated using the following cut-off points: ≤27 = low level of resilience and ≥36 = high level of resilience [43].

### 2.1. Ethical Considerations

The research project was evaluated and approved by the Ethics and Research Committee of the University Center for Health Science of the Universidad de Guadalajara (Mexico), with folio number CI-01520. The study was conducted according to the guidelines of the Declaration of Helsinki. All participants included in the study voluntarily provided their informed consent after reading the purposes of the study. Data are stored in a locked and password-protected computer under the principal investigator’s safekeeping to maintain confidentiality.

### 2.2. Statistical Analysis

The data obtained were analyzed with the SPSS v.23.0 statistical package (IBM Corp, Armonk, NY, USA). The significance level was set at α < 0.05. First, preliminary ANOVA was performed to examine whether any of the sociodemographic variables were related to the mental health variables (i.e., the impact of the event, anxiety, depression, and stress). Second, descriptive statistics were calculated for the sociodemographic variables (frequencies and percentages) and the study variables (mean and SD). Third, the relationship between the COVID-19 disease process variables (i.e., number of symptoms, relapses, isolation with or without family support, hospitalization, and oxygen use), the mental health variables (i.e., the impact of the event, depression, stress, and anxiety), and resilience was examined. In the case of continuous independent variables (i.e., number of symptoms and resilience), the relationship was examined using Pearson’s bivariate correlations. In the case of categorical independent variables (i.e., relapses, isolation with or without family support, hospitalization, and oxygen use), a one-factor ANOVA was performed, including each mental health indicator as a dependent variable. In the ANOVA, η^2^_p_ was included to estimate the effect size, defined as small (η^2^_p_ > 0.10), medium (η^2^_p_ > 0.25), and large (η^2^_p_ > 0.40) effects [44]. Finally, moderation analyses were conducted by multivariate regressions with the PROCESS 3.7 macro [45] for SPSS. Model 1 was constructed to study the moderating effect of resilience (moderator) on the relationship between the COVID-19 disease process variables (independent) and the mental health variables (dependent). Gender, healthcare worker/professional, and chronic illness were included as covariates to control their effect. The simple slopes of the variables involved in the moderation were plotted with the mean ±1 DT of the moderator.

## 3. Results

### 3.1. Description of the COVID-19 Survivors

The sample consisted of 253 survivors of COVID-19 in Mexico. Table 1 shows the general characteristics of the sample. The mean age of the participants was 35.97 years (SD = 10.61 years), ranging from 18 to 75 years, with the majority being young adults aged 18–39 years (65.2%) and female (68.8%). The sample had a high educational level, as approximately 75% of the participants reported having university education (undergraduate, graduate, and/or doctorate). More than 60% of the participants reported being health workers/professionals. A total of 23.3% reported the presence of previous chronic diseases, the most frequent being obesity and hypertension.

### 3.2. Physical and Mental Health Status of the COVID-19 Survivors

The means and correlations between the continuous variables are shown in Table 2. Most participants (62.1%) reported some level of distress, with 36% reporting having experienced a severe impact due to the event.

Between 13% and 35.2% of the survivors experienced anxious, depressive, or stress symptoms, with the highest prevalence of moderate (15%) or severe (4%) symptoms in the case of anxiety. Having more symptoms or having symptoms for more days was related to greater traumatic impact, anxiety, depression, and stress. In contrast, resilience showed a significant negative relationship with traumatic impact, depression, anxiety, and stress.

### 3.3. Physical and Mental Health of the COVID-19 Survivors

Preliminary ANOVA was performed between the sociodemographic and mental health variables. The results indicated that being a woman was associated with a greater impact of the event (*F* = 4.74, *p* < 0.01, η^2^_p_ = 0.019) and greater anxiety (*F* = 5.99, *p* < 0.01, η^2^_p_ = 0.023), whereas having an occupation other than health care worker/professional was associated with greater levels of depression (*F* = 5.47, *p* < 0.01, η^2^_p_ = 0.022), anxiety (*F* = 4.19, *p* < 0.01, η^2^_p_ = 0.017), and stress (*F* = 8.95, *p* < 0.001, η^2^_p_ = 0.035).

With respect to the categorical variables of the COVID-19 disease process (Table 3), most of the participants did not require hospitalization or oxygen. Almost all participants had family support during the isolation period (92.1%) and 15% experienced relapses. Not having support during isolation was associated with higher levels of depression and anxiety. Likewise, suffering relapses was the variable with the largest effect size in relation to the impact of the event, anxiety, depression, and stress. Neither hospitalization nor oxygen use was related to mental health.

### 3.4. Resilience Moderates the Effect of the Disease Process on Survivor Mental Health

Whether resilience moderates the effect of the COVID-19 disease process on mental health was examined (Table 4). Resilience was negatively associated with all mental health indicators. The interaction between resilience and family support during isolation, hospitalization, or oxygen use had no relationship with mental health.

**Figure 1:**
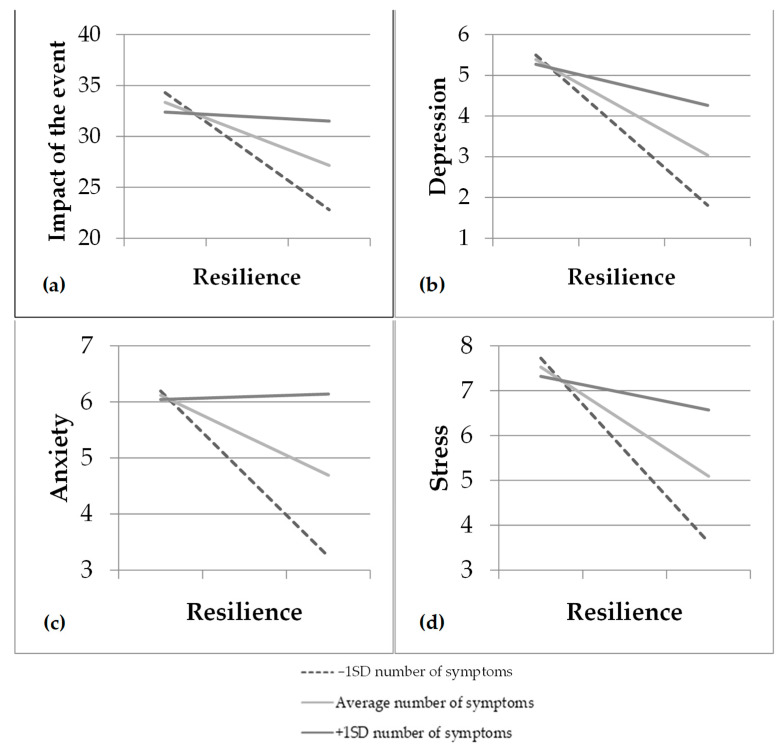
(**a**) Moderation of resilience on the traumatic impact of the event as a function of the number of symptoms of COVID-19; (**b**) moderation of resilience on depression as a function of the number of symptoms of COVID-19; (**c**) moderation of resilience on anxiety as a function of the number of symptoms of COVID-19; (**d**) moderation of resilience on stress as a function of the number of symptoms of COVID-19.

The interaction between the number of symptoms and resilience had a significant effect on all mental health indicators. As shown in Table 4, higher resilience was significantly related to lower impact of the event, stress, anxiety, and depression when the number of symptoms was low (≤4 symptoms) (impact of the event: *t* = −3.14, *p* = 0.002; depression: *t* = −4.00, *p* < 0.001; anxiety: *t* = −3.41, *p* < 0.001; stress: *t* = −4.40, *p* < 0.001) or medium (~7 symptoms) (impact of the event: *t* = −2.35, *p* = 0.019; depression: *t* = −3.56, *p* < 0.001; anxiety: *t* = −2.30, *p* = 0.022; stress: *t* = −3.63, *p* < 0.001), whereas it was not related to any mental health indicator when the number of symptoms was high (≥10 symptoms) (impact of the event: *t* = −3.14, *p* = 0.002; depression: *t* = −4.00, *p* < 0.001; anxiety: *t* = −3.41, *p* < 0.001; stress: *t* = −4.40, *p* < 0.001) (Figure 1).

**Figure 2:**
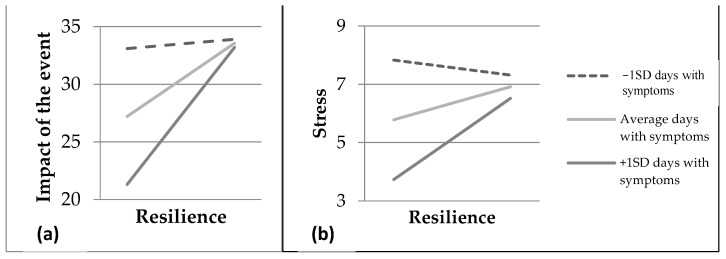
(**a**) Moderation of resilience on the traumatic impact of the event as a function of the number of days with COVID-19; (**b**) moderation of resilience on stress as a function of the number of days with COVID-19.

In terms of the number of days with COVID-19, we found that the interaction between disease duration and resilience had a similar effect on the impact of the event, and stress. The simple slopes indicated that resilience was related to a lower impact of the event and stress when the person had a short (≤3 days) (impact of the event: *t* = −3.05, *p* = 0.003; stress: *t* = −4.17, *p* < 0.001) or medium disease process (~9 days; impact of the event: *t* = −2.37, *p* = 0.019; stress: *t* = −3.65, *p* < 0.001), but not a prolonged one (≥16 days; impact of the event: *t* = −0.22, *p* = 0.828; stress: *t* = −0.97, *p* = 0.332). (Figure 2).

**Figure 3:**
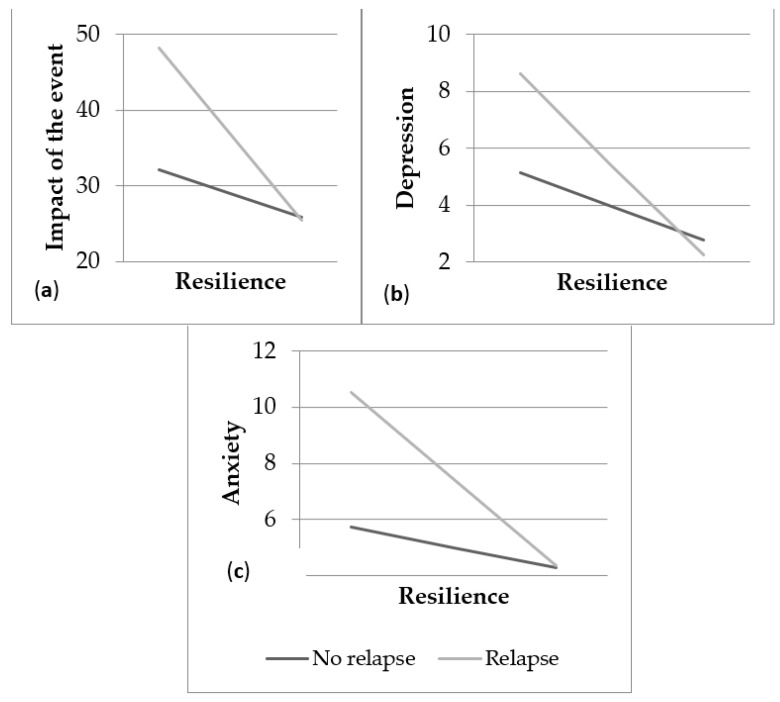
(**a**) Moderation of resilience on the traumatic impact of the event as a function of the presence of relapses; (**b**) moderation of resilience on depression as a function of the presence of relapses; (**c**) moderation of resilience on anxiety as a function of the presence of relapses.

Resilience was related to a lesser impact of the event, depression, and anxiety even in the presence of relapses. Relapses had more influence on the level of impact of the event, depression, and anxiety when resilience was lower. Specifically, the results of the simple slopes show that resilience was more strongly related to lower impact of the event, depression, and anxiety for those who experienced relapses (impact of the event: *t* = −3.35, *p* < 0.001; depression: *t* = −3.67, *p* < 0.001; anxiety: *t* = −3.86, *p* < 0.001) than for those who did not (*t* = −2.38, *p* = 0.018; depression: *t* = −3.58, *p* < 0.001; anxiety: *t* = −2.33, *p* = 0.021) (Figure 3).

## 4. Discussion

The complex and long-lasting pandemic and the events resulting from it, such as lockdowns, economic losses, isolation, and the fear of contagion, are major stressors amongst populations. Although most people recover from infection, a considerable proportion experience the disease process as a traumatic event [46,47]; moreover, meta-analyses have estimated that approximately half of survivors experience post-COVID-19 symptoms or long COVID-19 [48], which constitute an additional burden on the mental health of these people.

Preliminary analysis showed that being a woman and having an occupation other than health care worker/professional is related to greater mental health deterioration among survivors. These results are in line with previous studies conducted with the general population and healthcare workers/professionals in which the highest risk of psychological distress was found among women [49,50]. A previously published meta-analysis found no differences in the prevalence of psychological consequences between healthcare workers/professionals and the general population in infected or suspected COVID-19 samples [51] or in non-infected samples [52]. Conversely, the meta-analysis by Sun et al. [50] reported a higher incidence of anxiety and depression among uninfected frontline healthcare workers/professionals. The lower psychological distress reported by healthcare workers/professionals could be due to their background and previous experiences, often witnessing life-threatening situations, compared to the general population.

Most of the participants in the present study were young adults (mean age = 35.97 years, SD = 10.61 years); several studies have reported greater effects on mental health in this age group [33,53]. Different studies have reported that the elderly are actually more resilient to anxiety, depression, and stress-related mental health disorders [54]. Alodhayani et al. [49] highlighted that increasing age appears to confer protection against distress, despite being the most vulnerable group to SARS-CoV-2 infection, probably because of the development of more efficient psychological coping and adaptability during COVID-19 [55,56].

The sample was composed of people with a high educational level; some studies have indicated that a high educational level could be a protective factor against depression, anxiety, and stress [57]. However, there is evidence that a high level of education may also predispose to mental health issues [49]. In the present study, this variable was not significantly related to mental health.

Taken together, these results help to identify subpopulations with increased vulnerability to mental health deterioration as a consequence of SARS-CoV-2 infection, and so could help guide efforts to develop targeted health promotion and prevention programs to prepare these individuals to cope with the psychological distress caused by the disease.

The results suggested that resilience is negatively and significantly associated with the impact of the event, depression, anxiety, and stress. These findings agree with the scientific literature about the role of resilience as a protective factor against psychological distress in the face of traumatic life events such as the present pandemic [58,59]. However, resilience was also negatively and significantly associated with two indicators of the disease process: the number of symptoms and the number of days with symptoms.

The ANOVA results underscored the importance of relapses that emerged, with a small effect size. At first, SARS-CoV-2 was expected to induce a monophasic disease; however, the increasingly common cases of clinical recurrence of COVID-19 symptoms [60,61,62] have led to an investigation into whether all suspected COVID-19 relapses are due to a prolonged positive status or reinfection with a new strain [63,64,65]. Studies have indicated that clinical relapse could be due to a low level of neutralizing antibodies [66], whereas others have hypothesized that disease recurrence is due to an inflammatory syndrome because of an inappropriate immune response [61]. Although no subsequent RT-PCR testing was documented in the cases of recurrence of symptoms in our study, these instances draw attention to the persistence of the infection [60].

Hospitalization and requiring supplemental oxygen were not related to mental health in this sample, in contrast to previous findings in which the severity of the disease was associated with a higher risk of post-traumatic stress symptomatology [15], anxiety, and depression [67,68]. This result is contrary to that obtained by Einvik et al. [16], who reported a higher prevalence of psychological distress in survivors who were hospitalized compared to those who were not.

Moderation analysis highlighted the relevance of resilience as a variable buffering the effects of mental health deterioration in the aftermath of SARS-CoV-2 infection. Relapse was positively associated with higher levels of anxiety, stress, and the impact of the event. In this sense, it was hypothesized that the recurrence of COVID-19 symptoms could be due to an inappropriate immune response [61]; additionally, it was reported that coronavirus infection produces perturbation of the immune system, which could induce psychological and psychiatric sequelae in survivors [8], linking the immune system function to COVID-19 psychological outcomes. However, more research is needed to confirm these findings and hypotheses.

Resilience buffered the effect of the number of COVID-19 symptoms on stress, as well as the effect of the number of days with the disease on stress, anxiety, and traumatic impact. However, the results from moderation models indicated that resilience may lose its protective effect on mental health when people suffer a greater number of COVID-19-related symptoms or when facing prolonged disease symptomology. Moreover, the level of resilience must be moderate or high to exert a protective effect.

Resilience was related to lower levels of impact of the event, depression, and stress, even in the presence of relapses. We found that people with high resilience show less anxiety when experiencing a relapse than people with medium or low levels of resilience. Similar findings were reported in noninfected populations from a multinational study, where an increase of one standard deviation in the resilience score was associated with reduced rates of anxiety and depression [31]. Nevertheless, the present study is, to the best of our knowledge, the first to investigate resilience as a personal factor moderating the effects of COVID-19 on the mental health of recovered patients.

This study has several limitations. First, it was a cross-sectional study, which did not allow inferences of causality in the results. In this sense, longitudinal research designs would be appropriate to delve deeper into survivors’ mental health trajectories following SARS-CoV-2 infection, as the timing of the assessment seems to influence the perception of psychological distress. Second, COVID-19 relapse is currently defined as the clinical recurrence of symptoms compatible with COVID-19 accompanied by a positive RT-PCR test within 90 days of primary infection and supported by the absence of exposure to the disease [69]. Unfortunately, in the present study, we were unable to access information on the participant´s subsequent RT-PCR testing. Moreover, most healthcare workers/professionals are compelled to return to their workplace soon enough to be re-exposed to SARS-CoV-2. Despite the lack of information about subsequent RT-PCR testing and the re-exposure of survivors, the impact of the recurrence of COVID-19 symptoms on the mental health of survivors was present and should not be overlooked. Future research should consider these criteria to advance our understanding of the psychological effects of reinfection and relapse.

Notwithstanding these limitations, the present study provides valuable data on the mental health of COVID-19 survivors and is one of the first studies to investigate the moderating role of resilience on the mental health of COVID-19 survivors in the region.

## 5. Conclusions

In summary, the results suggest that resilience has an important protective role in coping with COVID-19, especially in coping with a relapse in symptoms. However, only when the number of symptoms was low or moderate did resilience reduce the impact of the event, depression, anxiety, and stress. Similarly, only when the duration of COVID-19 was short and resilience levels were medium or high was psychological distress reduced, emphasizing that personal psychological resources may not be sufficient for people when facing severe disease and for those experiencing a long illness duration. Timely psychological counseling and interventions to provide information on coping strategies should be implemented in the general population and, specifically, targeting identified subpopulations with a higher risk of mental health deterioration, i.e., women, and young people. Further research is needed on psychological and social resources to reduce the mental health impact of more severe and long COVID-19.

## Figures and Tables

**Table 1:** Sociodemographic data of the survivors (n = 253).

Variable	n (%)
Gender	
Woman	174 (68.8)
Man	79 (31.2)
Age group	
18–39	165 (65.2)
40–59	84 (33.2)
60–75	4 (1.6)
Educational level	
Basic education	62 (24.7)
Bachelor’s degree	152 (60.6)
Master’s degree or higher	37 (14.7)
Occupation	
Housekeeper	9 (3.6)
Worker	10 (4.0)
Trader	2 (0.8)
Employee	8 (3.2)
Professional	80 (31.6)
Student	122 (48.2)
Other occupations	22 (8.7)
Healthcare worker/professional	
Yes	156 (63.4)
No	90 (36.6)
Relationships	
In a stable relationship	177 (70.0)
In an unstable relationship	11 (4.3)
Single	65 (25.7)
Children	
Has children under 16	103 (40.7)
Has children over 16	43 (17.0)
No children	107 (42.3)
Household size	
One person	18 (7.1)
Two people	35 (13.8)
Three people	83 (32.8)
Four people	62 (24.5)
Five or more people	55 (21.7)
With a family member over 60	
Yes	181 (71.5)
No	72 (28.5)
Medical insurance	
Social security	173 (68.4)
Private medical expenses	33 (13.0)
Without coverage	47 (18.6)
Chronic illness	
Yes	59 (23.3)
No	194 (76.7)
Type of chronic illness	
Hypertension	21 (8.3)
Obesity	27 (10.7)
Diabetes	14 (5.5)
Cancer	3 (1.2)
Chronic kidney disease	3 (1.2)

**Table 2:** Median, standard deviation, and correlations between the variables of study.

Variable		Correlations					
	M ± SD	1	2	3	4	5	6
1. COVID-19-related symptoms	7.15 ± 3.36						
2. Days with symptoms	9.30 ± 6.41	0.36 ***					
3. Impact of the event	30.31 ± 18.57	0.23 ***	0.24 ***				
4. Depression	4.17 ± 4.61	0.22 **	0.17 **	0.69 ***			
5. Anxiety	5.39 ± 4.64	0.31 ***	0.30 ***	0.74 ***	0.77 ***		
6. Stress	6.28 ± 4.90	0.24 ***	0.21 ***	0.80 ***	0.84 ***	0.85 ***	
7. Resilience	29.46 ± 6.64	−0.03	−0.08	−0.19 **	−0.27 ***	−0.19 **	−0.22 ***

Note: ** *p* < 0.01, *** *p* < 0.001.

**Table 3:** Mental health of the COVID-19 survivors as a function of the disease.

		Impact of the Event				Depression				Anxiety				Stress			
Variable	n	M	SD	*F*	*η* ^2^_p_	M	SD	*F*	*η* ^2^_p_	M	SD	*F*	*η* ^2^_p_	M	SD	*F*	*η* ^2^_p_
Support during isolation				1.16	0.005			4.50 *	0.018			4.99 *	0.020			3.36	0.013
Isolated without family support	20	34.60	20.05			6.25	6.01			7.60	5.57			8.20	5.92		
Isolated with family support	233	29.94	18.44			3.99	4.44			5.20	4.52			6.12	4.78		
Relapses				10.61 **	0.041			6.67 *	0.026			18.26 ***	0.068			12.34 **	0.047
Yes	40	38.92	23.32			5.87	5.31			8.18	5.56			8.73	5.10		
No	213	28.69	17.13			3.85	4.41			4.87	4.27			5.83	4.73		
Hospitalization				2.56	0.010			0.40	0.002			2.88 *^t^*	0.011			1.027	0.004
Yes	14	38.00	22.62			4.93	3.67			7.43	5.23			7.57	4.55		
No	239	29.86	28.26			4.12	4.66			5.27	4.59			6.21	4.91		
Oxygen				2.05	0.008			1.32	0.005			3.71	0.015			0.93	0.004
Yes	20	36.00	19.56			5.30	4.58			7.30	4.68			7.30	4.59		
No	233	29.82	18.45			4.07	4.61			5.23	4.61			6.20	4.92		

Note: *^t^* tendency, * *p* < 0.05, ** *p* < 0.01, *** *p* < 0.001.

**Table 4:** Multivariate Regression Models of Mental health of the COVID-19 survivors as a function of disease severity and resilience.

	Impact of the Event			Depression			Anxiety			Stress		
Independent Variable	*β*	SE	*r* ^2^	*β*	SE	*r* ^2^	*β*	SE	*r* ^2^	*β*	SE	*r* ^2^
			0.23			0.22			0.30			0.28
Resilience	−0.46 *	0.19		−0.17 ***	0.05		−0.11 *	0.05		−0.18 ***	0.05	
Number of symptoms	0.51	0.37		0.17	0.09		0.21 *	0.09		0.19 *	0.09	
Days with symptoms	0.49 **	0.19		0.05	0.05		0.14 **	0.04		0.09	0.05	
Isolation without family support	5.43	4.22		2.70 *	1.05		2.83 **	0.99		2.79 **	1.07	
Relapses	7.84 *	3.12		1.43	0.79		2.44 **	0.74		2.08 **	0.79	
Hospitalization	10.76	6.56		−0.74	1.64		1.09	1.54		0.89	1.67	
Oxygen	−3.54	5.66		0.80	1.42		0.85	1.34		−0.12	1.44	
Resilience × Number of symptoms	0.12 *	0.05		0.03 *	0.01		0.03 *	0.01		0.04 *	0.01	
Resilience × Days with symptoms	0.07 *	0.03		0.01	0.01		0.01	0.01		0.02 **	0.01	
Resilience × Isolation without family support	−0.45	0.53		0.04	0.13		−0.01	0.13		0.10	0.14	
Resilience × Relapses	−1.23 *	0.03		−0.29 *	0.13		−0.35 **	0.13		−0.24	0.14	
Resilience × Hospitalization	−0.92	1.12		0.27	0.28		0.25	0.26		−0.01	0.28	
Resilience × Oxygen	1.63	1.04		−0.16	0.26		−0.09	0.24		0.15	0.26	

Note: Independent variable; interaction = resilience X COVID-19 disease process variable. * *p* < 0.05, ** *p* < 0.01, *** *p* < 0.001.

## Data Availability

The data presented in this study are available on request from the corresponding author. The data are not publicly available due to the protection of personal data that could compromise the privacy of research participants.
